# Supplementary material for: Prognostic Ability of Enhancer RNAs in Metastasis of Non-Small Cell Lung Cancer
Source: Molecules. 2022 Jun 26;27(13):4108. doi: 10.3390/molecules27134108 (PMC9268450; doi:10.3390/molecules27134108)
Supplement: Supplementary file 1 [file molecules-27-04108-s001.zip › Table S1.pdf]

Table S1. The target genes and drug response information of eRNAs.

The target genes information of ANXA8L1 (ENSR00000027547)

Cancer Type	Enhancer Id	Mid Location	Target Gene	Gene Location	TSS	Distance	Rs	-Log10FDR
KIRP	ENSR00000027547	10:46363987	AGAP4	10:45853875-45825594	10	538393	0.9006	>15
CHOL	ENSR00000027547	10:46363987	AGAP4	10:45853875-45825594	10	538393	0.7653	2.382482668
KIRP	ENSR00000027547	10:46363987	GPRIN2	10:46555530-46549044	10	185057	0.6136	>15
THYM	ENSR00000027547	10:46363987	AGAP4	10:45853875-45825594	10	538393	0.5597	>15
MESO	ENSR00000027547	10:46363987	AGAP4	10:45853875-45825594	10	538393	0.4853	3.21925102

KIRP: Kidney renal papillary cell carcinoma.; CHOL: cholangiocarcinoma; THYM: Thymoma; MESO: Mesothelioma.

The target genes information of CASTOR2 (ENSR00000326772)

Cancer Type	Enhancer Id	Mid Location	Target Gene	Gene Location	TSS	Distance	Rs	-Log10FDR
GBM	ENSR00000326772	7:74933000	SPDYE5	7:75493625-75504304	7	571304	0.6936	>15
GBM	ENSR00000326772	7:74933000	NSUN5P1	7:75410322-75416787	7	483787	0.5987	>15
GBM	ENSR00000326772	7:74933000	POM121C	7:75486271-75416787	7	483787	0.5017	7.775839251
GBM	ENSR00000326772	7:74933000	GTF2IRD1	7:74453790-74602604	7	330396	0.4995	7.679481017
GBM	ENSR00000326772	7:74933000	HIP1	7:75738962-75533300	7	600300	0.4755	6.820171556
GBM	ENSR00000326772	7:74933000	GTF2IRD2	7:74851551-74796144	7	136856	0.4142	5.079213164
GBM	ENSR00000326772	7:74933000	STAG3L1	7:75359194-75395383	7	462383	0.3807	4.269664945
GBM	ENSR00000326772	7:74933000	CLIP2	7:74289475-74405943	7	527057	0.3572	3.753339682
GBM	ENSR00000326772	7:74933000	GTF2IRD2B	7:75092573-75149817	7	216817	0.3488	3.621471434
GBM	ENSR00000326772	7:74933000	STAG3L2	7:74890610-74882163	7	50837	0.3388	3.376917376
GBM	ENSR00000326772	7:74933000	GTF2IP1	7:75237696-75185385	7	252385	0.3331	3.264306714
GBM	ENSR00000326772	7:74933000	GTF2I	7:74657667-74760692	7	172308	0.3282	3.169741516

GBM: Glioblastoma multiforme.

The target genes information of CYP4B1 (ENSR00000251622)

Cancer Type	Enhancer Id	Mid Location	Target Gene	Gene Location	TSS	Distance	Rs	-Log10FDR
BRCA	ENSR00000251622	1:46851100	CYP4Z2P	1:46900437-46843095	1	8005	0.8972	>15
BLCA	ENSR00000251622	1:46851100	CYP4B1	1:46757838-46819413	1	31687	0.8601	>15
OV	ENSR00000251622	1:46851100	CYP4Z2P	1:46900437-46843095	1	8005	0.8397	>15
LUAD	ENSR00000251622	1:46851100	CYP4B1	1:46757838-46819413	1	31687	0.8072	>15
LUAD	ENSR00000251622	1:46851100	CYP4Z2P	1:46900437-46843095	1	8005	0.7848	>15
OV	ENSR00000251622	1:46851100	CYP4B1	1:46757838-46819413	1	31687	0.749	>15
BLCA	ENSR00000251622	1:46851100	CYP4Z2P	1:46900437-46843095	1	8005	0.7482	>15
BRCA	ENSR00000251622	1:46851100	CYP4Z1	1:47067488-47118319	1	267219	0.7169	>15
LUAD	ENSR00000251622	1:46851100	CYP4Z1	1:47067488-47118319	1	267219	0.6627	>15
OV	ENSR00000251622	1:46851100	CYP4Z1	1:47067488-47118319	1	267219	0.6285	>15
BLCA	ENSR00000251622	1:46851100	CYP4Z1	1:47067488-47118319	1	267219	0.6191	>15
BRCA	ENSR00000251622	1:46851100	CYP4X1	1:47023568-47050751	1	199651	0.6027	>15
BRCA	ENSR00000251622	1:46851100	CYP4A22	1:47137435-47149741	1	298641	0.6021	>15
LUAD	ENSR00000251622	1:46851100	CYP4A11	1:46941484-46929177	1	78077	0.5506	>15
BRCA	ENSR00000251622	1:46851100	CYP4A11	1:46941484-46929177	1	78077	0.5451	>15
BLCA	ENSR00000251622	1:46851100	CYP4A11	1:46941484-46929177	1	78077	0.5403	>15
OV	ENSR00000251622	1:46851100	CYP4A22	1:47137435-47149741	1	298641	0.5274	>15
BLCA	ENSR00000251622	1:46851100	FAAH	1:46394265-46413848	1	437252	0.5105	>15
LUAD	ENSR00000251622	1:46851100	CYP4X1	1:47023568-47050751	1	199651	0.4948	>15
BRCA	ENSR00000251622	1:46851100	CYP4B1	1:46757838-46819413	1	31687	0.486	>15
LUAD	ENSR00000251622	1:46851100	CYP4A22	1:47137435-47149741	1	298641	0.4655	>15
LUAD	ENSR00000251622	1:46851100	FAAH	1:46394265-46413848	1	437252	0.458	>15
BLCA	ENSR00000251622	1:46851100	CYP4X1	1:47023568-47050751	1	199651	0.4212	>15
OV	ENSR00000251622	1:46851100	CYP4A11	1:46941484-46929177	1	78077	0.4154	9.206239819
BLCA	ENSR00000251622	1:46851100	CYP4A22	1:47137435-47149741	1	298641	0.4109	>15
LUAD	ENSR00000251622	1:46851100	ATPAF1	1:46673867-46632737	1	218363	0.3771	>15
LUAD	ENSR00000251622	1:46851100	TAL1	1:47232220-47216290	1	365190	0.371	>15
BRCA	ENSR00000251622	1:46851100	PIK3R3	1:46133036-46040140	1	810960	0.3471	>15
OV	ENSR00000251622	1:46851100	ATPAF1	1:46673867-46632737	1	218363	0.3453	6.174023683
BRCA	ENSR00000251622	1:46851100	TSPAN1	1:46175073-46185958	1	665142	0.3109	>15
BLCA	ENSR00000251622	1:46851100	PIK3R3	1:46133036-46040140	1	810960	0.3023	8.147798935

BRCA: Breast invasive carcinoma; BLCA: Bladder Urothelial Carcinoma; OV: Ovarian serous cystadenocarcinoma; LUAD: Lung adenocarcinoma.

The target genes information of GTF2H2C (ENSR00000182109)

Cancer Type	Enhancer Id	Mid Location	Target Gene	Gene Location	TSS	Distance	Rs	-Log10FDR
LGG	ENSR00000182109	5:71097436	NAIP	5:71025114-70968483	5	128953	0.4008	>15
LGG	ENSR00000182109	5:71097436	SERF1A	5:70900665-70918530	5	178906	0.307	10.60745428

LGG: Brain Lower Grade Glioma.

The target genes information of PSMF1 (20:1141246-1147246)

Cancer Type	Enhancer Id	Mid Location	Target Gene	Gene Location	TSS	Distance	Rs	-Log10FDR
STAD	20:1141246-1147246	20:1144246	STK35	20:2101611-2177038	20	957365	0.4502	0
STAD	20:1141246-1147246	20:1144246	CSNK2A1	20:543821-473591	20	600425	0.4432	0
STAD	20:1141246-1147246	20:1144246	TBC1D20	20:462553-435480	20	681693	0.4073	0
STAD	20:1141246-1147246	20:1144246	ZCCHC3	20:296968-300321	20	847278	0.3733	3.290282e-13
STAD	20:1141246-1147246	20:1144246	C20orf96	20:290778-270863	20	853468	0.3417	5.24085e-11

STAD: Stomach adenocarcinoma.

The target genes information of TNS4 (17:40489449-40495449)

Cancer Type	Enhancer Id	Mid Location	Target Gene	Gene Location	TSS	Distance	Rs	-Log10FDR
STAD	17:40489449-40495449	17:40492449	TNS4	17:40501597-40475828	17	9148	0.8784	0
PAAD	17:40489449-40495449	17:40492449	TNS4	17:40501597-40475828	17	9148	0.8657	7.59220619713681e-54
READ	17:40489449-40495449	17:40492449	TNS4	17:40501597-40475828	17	9148	0.8391	0
CESC	17:40489449-40495449	17:40492449	TNS4	17:40501597-40475828	17	9148	0.83	1.79690941916321e-77
BLCA	17:40489449-40495449	17:40492449	TNS4	17:40501597-40475828	17	9148	0.8231	1.77998008881277e-104
LUSC	17:40489449-40495449	17:40492449	TNS4	17:40501597-40475828	17	9148	0.7857	6.26755584042288e-115
HNSC	17:40489449-40495449	17:40492449	TNS4	17:40501597-40475828	17	9148	0.5721	0
LUSC	17:40489449-40495449	17:40492449	RAPGEFL1	17:40177010-40195656	17	315439	0.5208	5.45289675206652e-38
READ	17:40489449-40495449	17:40492449	MSL1	17:40122298-40136916	17	370151	0.5205	6.32969302481917e-06
BLCA	17:40489449-40495449	17:40492449	RAPGEFL1	17:40177010-40195656	17	315439	0.472	2.95812800710651e-23
PAAD	17:40489449-40495449	17:40492449	ERBB2	17:39687914-39730426	17	804535	0.411	2.96265881875012e-07
LUSC	17:40489449-40495449	17:40492449	KRT34	17:41382403-41377650	17	889954	0.4107	2.59633134512955e-22
READ	17:40489449-40495449	17:40492449	GSDMA	17:39953263-39977766	17	539186	0.4062	0.00092652
PAAD	17:40489449-40495449	17:40492449	GRB7	17:39737927-39747291	17	754522	0.3939	1.08575412727218e-06
PAAD	17:40489449-40495449	17:40492449	GSDMB	17:39919854-39904595	17	572595	0.3926	1.19281400967078e-06
PAAD	17:40489449-40495449	17:40492449	NR1D1	17:40100725-40092787	17	391724	0.3842	2.19612400321798e-06
PAAD	17:40489449-40495449	17:40492449	RAPGEFL1	17:40177010-40195656	17	315439	0.3803	2.88972264482997e-06
LUSC	17:40489449-40495449	17:40492449	NR1D1	17:40100725-40092787	17	391724	0.3788	8.51558355139561e-19
READ	17:40489449-40495449	17:40492449	CDC6	17:40287633-40304657	17	204816	0.3788	0.002350528
LUSC	17:40489449-40495449	17:40492449	MSL1	17:40122298-40136916	17	370151	0.3705	5.9775685551399e-18
READ	17:40489449-40495449	17:40492449	TOP2A	17:40417950-40388516	17	74499	0.3518	0.005426871
READ	17:40489449-40495449	17:40492449	KRTAP9-4	17:41249687-41250653	17	757238	0.3384	0.007602445
BLCA	17:40489449-40495449	17:40492449	KRT31	17:41397592-41393724	17	905143	0.3381	1.47726101059421e-11
CESC	17:40489449-40495449	17:40492449	RAPGEFL1	17:40177010-40195656	17	315439	0.3347	5.45541270606501e-08
LUSC	17:40489449-40495449	17:40492449	KRT33B	17:41369800-41363494	17	877351	0.3328	1.75545380533646e-14
HNSC	17:40489449-40495449	17:40492449	MSL1	17:40122298-40136916	17	370151	0.3228	4.10647177862099e-13
LUSC	17:40489449-40495449	17:40492449	KRT10	17:40822595-40818117	17	330146	0.322	1.36795637316005e-13
READ	17:40489449-40495449	17:40492449	KRTAP1-5	17:41027202-41026026	17	534753	0.3203	0.012618281
LUSC	17:40489449-40495449	17:40492449	KRT33A	17:41350812-41346092	17	858363	0.3089	1.5083559474837e-12
LUSC	17:40489449-40495449	17:40492449	KRT31	17:41397592-41393724	17	905143	0.3019	5.18743177014694e-12
PAAD	17:40489449-40495449	17:40492449	TOP2A	17:40417950-40388516	17	74499	0.3011	0.000356553

STAD: Stomach adenocarcinoma; PAAD: Pancreatic adenocarcinoma; READ: Rectum adenocarcinoma; CESC: Cervical squamous cell carcinoma and endocervical adenocarcinoma; BLCA: Bladder Urothelial Carcinoma; LUSC: Lung squamous cell carcinoma; HNSC: Head and Neck squamous cell carcinoma.

The drug response information of CASTOR2 (ENSR00000326772)

Id	Location	Drug	Rs	FDR	Database
ENSR00000326772	7:74930000-74936000	Docetaxel	0.3042	0	GDSC
ENSR00000326772	7:74930000-74936000	selumetinib	0.244	0.0001	GDSC
ENSR00000326772	7:74930000-74936000	Trametinib	0.243	0.0001	GDSC
ENSR00000326772	7:74930000-74936000	PD-0325901	0.2415	0.0003	GDSC
ENSR00000326772	7:74930000-74936000	RDEA119	0.233	0.0002	GDSC
ENSR00000326772	7:74930000-74936000	RDEA119	0.2248	0.0007	GDSC
ENSR00000326772	7:74930000-74936000	JNJ-26854165	0.217	0.001	GDSC
ENSR00000326772	7:74930000-74936000	17-AAG	0.2075	0.0026	GDSC
ENSR00000326772	7:74930000-74936000	GSK1904529A	0.2004	0.0032	GDSC
ENSR00000326772	7:74930000-74936000	AMG-706	0.1968	0.0056	GDSC
ENSR00000326772	7:74930000-74936000	Elesclomol	0.1933	0.0112	GDSC
ENSR00000326772	7:74930000-74936000	JNK Inhibitor VIII	0.1928	0.0064	GDSC
ENSR00000326772	7:74930000-74936000	CHIR-99021	0.1922	0.0096	GDSC
ENSR00000326772	7:74930000-74936000	Bleomycin (50 uM)	0.1881	0.0038	GDSC
ENSR00000326772	7:74930000-74936000	AP-24534	0.1848	0.0404	GDSC
ENSR00000326772	7:74930000-74936000	CI-1040	0.1766	0.0118	GDSC
ENSR00000326772	7:74930000-74936000	AZD6482	0.1744	0.0359	GDSC
ENSR00000326772	7:74930000-74936000	HG-6-64-1	0.1693	0.0213	GDSC
ENSR00000326772	7:74930000-74936000	Temsirolimus	0.1671	0.029	GDSC
ENSR00000326772	7:74930000-74936000	FTI-277	0.161	0.0273	GDSC
ENSR00000326772	7:74930000-74936000	XAV939	0.1599	0.0185	GDSC
ENSR00000326772	7:74930000-74936000	Linifanib	0.1529	0.0391	GDSC
ENSR00000326772	7:74930000-74936000	CCT007093	0.151	0.0392	GDSC
ENSR00000326772	7:74930000-74936000	piperlongumine	0.1509	0.027	GDSC
ENSR00000326772	7:74930000-74936000	BMS-708163	0.1484	0.0433	GDSC
ENSR00000326772	7:74930000-74936000	(5Z)-7-Oxozeaenol	0.1461	0.0322	GDSC
ENSR00000326772	7:74930000-74936000	I-BET-762	-0.149	0.0426	GDSC
ENSR00000326772	7:74930000-74936000	CAY10603	-0.1559	0.0311	GDSC
ENSR00000326772	7:74930000-74936000	BMS345541	-0.1581	0.0224	GDSC
ENSR00000326772	7:74930000-74936000	CUDC-101	-0.1596	0.034	GDSC
ENSR00000326772	7:74930000-74936000	XMD13-2	-0.1682	0.021	GDSC
ENSR00000326772	7:74930000-74936000	KIN001-102	-0.1789	0.0065	GDSC
ENSR00000326772	7:74930000-74936000	MP470	-0.1796	0.0192	GDSC
ENSR00000326772	7:74930000-74936000	NPK76-II-72-1	-0.1811	0.0068	GDSC
ENSR00000326772	7:74930000-74936000	Vorinostat	-0.1852	0.011	GDSC
ENSR00000326772	7:74930000-74936000	Belinostat	-0.1869	0.0087	GDSC
ENSR00000326772	7:74930000-74936000	LAQ824	-0.1955	0.0039	GDSC
ENSR00000326772	7:74930000-74936000	TAK-715	-0.1959	0.0029	GDSC
ENSR00000326772	7:74930000-74936000	AR-42	-0.2077	0.0032	GDSC

The drug response information of CYP4B1 (ENSR00000251622)

Id	Location	Drug	Rs	FDR	Database
ENSR00000251622	1:46848100-46854100	HG-5-113-01	0.3187	0.0019	GDSC
ENSR00000251622	1:46848100-46854100	Foretinib	0.2717	0.0001	GDSC
ENSR00000251622	1:46848100-46854100	AZD7762	0.253	0.0004	GDSC
ENSR00000251622	1:46848100-46854100	RDEA119	0.2202	0.0004	GDSC
ENSR00000251622	1:46848100-46854100	MPS-1-IN-1	0.2158	0.0039	GDSC
ENSR00000251622	1:46848100-46854100	selumetinib	0.2149	0.0008	GDSC
ENSR00000251622	1:46848100-46854100	Trametinib	0.1995	0.0015	GDSC
ENSR00000251622	1:46848100-46854100	AP-24534	0.1936	0.0376	GDSC
ENSR00000251622	1:46848100-46854100	PD-0332991	0.1831	0.0163	GDSC
ENSR00000251622	1:46848100-46854100	BX-795	0.177	0.0323	GDSC
ENSR00000251622	1:46848100-46854100	Bleomycin (50 uM)	0.1761	0.0071	GDSC
ENSR00000251622	1:46848100-46854100	CEP-701	0.1747	0.0415	GDSC
ENSR00000251622	1:46848100-46854100	Camptothecin	0.1724	0.0331	GDSC
ENSR00000251622	1:46848100-46854100	RDEA119	0.1695	0.0134	GDSC
ENSR00000251622	1:46848100-46854100	HG-6-64-1	0.1679	0.0225	GDSC
ENSR00000251622	1:46848100-46854100	CI-1040	0.1671	0.0176	GDSC
ENSR00000251622	1:46848100-46854100	TPCA-1	0.1614	0.0182	GDSC
ENSR00000251622	1:46848100-46854100	BMS-754807	0.1583	0.038	GDSC
ENSR00000251622	1:46848100-46854100	Afatinib	-0.1329	0.0395	GDSC
ENSR00000251622	1:46848100-46854100	OSI-930	-0.1594	0.0443	GDSC
ENSR00000251622	1:46848100-46854100	WZ3105	-0.175	0.0083	GDSC
ENSR00000251622	1:46848100-46854100	THZ-2-49	-0.1772	0.0085	GDSC
ENSR00000251622	1:46848100-46854100	KIN001-102	-0.1829	0.0054	GDSC
ENSR00000251622	1:46848100-46854100	Afatinib	-0.2128	0.0013	GDSC

The drug response information of GTF2H2C (ENSR00000182109)

Id	Location	Drug	Rs	FDR	Database
ENSR00000182109	5:71094436-71100436	Gefitinib	0.3054	7.8057E-06	GDSC
ENSR00000182109	5:71094436-71100436	Afatinib	0.2884	8.6218E-06	GDSC
ENSR00000182109	5:71094436-71100436	Bosutinib	0.2744	0.0023	GDSC
ENSR00000182109	5:71094436-71100436	Afatinib	0.2617	0	GDSC
ENSR00000182109	5:71094436-71100436	Veliparib	0.2328	0.0009	GDSC
ENSR00000182109	5:71094436-71100436	Docetaxel	0.2294	0.0008	GDSC
ENSR00000182109	5:71094436-71100436	XAV939	0.2255	0.0007	GDSC
ENSR00000182109	5:71094436-71100436	PD-0325901	0.2251	0.0008	GDSC
ENSR00000182109	5:71094436-71100436	JNK Inhibitor VIII	0.2221	0.0013	GDSC
ENSR00000182109	5:71094436-71100436	CCT007093	0.2135	0.0019	GDSC
ENSR00000182109	5:71094436-71100436	JNJ-26854165	0.2109	0.0013	GDSC
ENSR00000182109	5:71094436-71100436	selumetinib	0.208	0.0012	GDSC
ENSR00000182109	5:71094436-71100436	Lenalidomide	0.2054	0.0098	GDSC
ENSR00000182109	5:71094436-71100436	selumetinib	0.1967	0.0063	GDSC
ENSR00000182109	5:71094436-71100436	RDEA119	0.1937	0.0041	GDSC
ENSR00000182109	5:71094436-71100436	RDEA119	0.1924	0.0024	GDSC
ENSR00000182109	5:71094436-71100436	Trametinib	0.1901	0.0026	GDSC
ENSR00000182109	5:71094436-71100436	17-AAG	0.1884	0.0073	GDSC
ENSR00000182109	5:71094436-71100436	CP724714	0.1812	0.0184	GDSC
ENSR00000182109	5:71094436-71100436	CI-1040	0.1803	0.01	GDSC
ENSR00000182109	5:71094436-71100436	Bicalutamide	0.1725	0.0199	GDSC
ENSR00000182109	5:71094436-71100436	Cetuximab	0.1681	0.0135	GDSC
ENSR00000182109	5:71094436-71100436	FTI-277	0.1585	0.0303	GDSC
ENSR00000182109	5:71094436-71100436	Bicalutamide	0.1562	0.0335	GDSC
ENSR00000182109	5:71094436-71100436	Bleomycin (50 uM)	0.1362	0.0412	GDSC
ENSR00000182109	5:71094436-71100436	BX-912	-0.1482	0.0434	GDSC
ENSR00000182109	5:71094436-71100436	TPCA-1	-0.1553	0.0242	GDSC
ENSR00000182109	5:71094436-71100436	Belinostat	-0.1613	0.0291	GDSC
ENSR00000182109	5:71094436-71100436	I-BET-762	-0.1626	0.024	GDSC
ENSR00000182109	5:71094436-71100436	CAY10603	-0.1914	0.0063	GDSC
ENSR00000182109	5:71094436-71100436	NPK76-II-72-1	-0.1921	0.0039	GDSC
ENSR00000182109	5:71094436-71100436	AR-42	-0.194	0.0059	GDSC

The drug response information of TNS4 (17:40489449-40495449)

Id	Location	Drug	Rs	FDR	Database
17:40489449-40495449	17:40489449-40495449	GSK269962A	0.2702	0	GDSC
17:40489449-40495449	17:40489449-40495449	TW 37	0.2456	0.0001	GDSC
17:40489449-40495449	17:40489449-40495449	GSK429286A	0.2366	0.0002	GDSC
17:40489449-40495449	17:40489449-40495449	CEP-701	0.2193	0.0018	GDSC
17:40489449-40495449	17:40489449-40495449	Camptothecin	0.2149	0.0024	GDSC
17:40489449-40495449	17:40489449-40495449	FK866	0.2087	0.0018	GDSC
17:40489449-40495449	17:40489449-40495449	UNC0638	0.2026	0.0022	GDSC
17:40489449-40495449	17:40489449-40495449	SN-38	0.1996	0.0027	GDSC
17:40489449-40495449	17:40489449-40495449	TL-1-85	0.1914	0.0042	GDSC
17:40489449-40495449	17:40489449-40495449	piperlongumine	0.1887	0.005	GDSC
17:40489449-40495449	17:40489449-40495449	BX-795	0.1869	0.0103	GDSC
17:40489449-40495449	17:40489449-40495449	PI-103	0.1847	0.0064	GDSC
17:40489449-40495449	17:40489449-40495449	AZD7762	0.1772	0.0157	GDSC
17:40489449-40495449	17:40489449-40495449	Vinblastine	0.169	0.0223	GDSC
17:40489449-40495449	17:40489449-40495449	(5Z)-7-Oxozeaenol	0.1676	0.0153	GDSC
17:40489449-40495449	17:40489449-40495449	TG101348	0.1657	0.0159	GDSC
17:40489449-40495449	17:40489449-40495449	BX-912	0.1623	0.0185	GDSC
17:40489449-40495449	17:40489449-40495449	Talazoparib	0.1587	0.0237	GDSC
17:40489449-40495449	17:40489449-40495449	Etoposide	0.1576	0.0249	GDSC
17:40489449-40495449	17:40489449-40495449	Vorinostat	0.1555	0.0384	GDSC
17:40489449-40495449	17:40489449-40495449	OSI-027	0.1542	0.0274	GDSC
17:40489449-40495449	17:40489449-40495449	Genentech Cpd 10	0.1538	0.0271	GDSC
17:40489449-40495449	17:40489449-40495449	Y-39983	0.1478	0.0351	GDSC
17:40489449-40495449	17:40489449-40495449	JW-7-24-1	0.1438	0.0416	GDSC
17:40489449-40495449	17:40489449-40495449	HG-6-64-1	0.142	0.0477	GDSC
17:40489449-40495449	17:40489449-40495449	GW-2580	-0.1414	0.0459	GDSC
17:40489449-40495449	17:40489449-40495449	EKB-569	-0.1428	0.0433	GDSC
17:40489449-40495449	17:40489449-40495449	BMS-708163	-0.1437	0.0418	GDSC
17:40489449-40495449	17:40489449-40495449	XAV939	-0.1467	0.039	GDSC
17:40489449-40495449	17:40489449-40495449	Bicalutamide	-0.1473	0.0437	GDSC
17:40489449-40495449	17:40489449-40495449	CH5424802	-0.1476	0.0363	GDSC
17:40489449-40495449	17:40489449-40495449	AKT inhibitor VIII	-0.1528	0.0307	GDSC
17:40489449-40495449	17:40489449-40495449	Lisitinib	-0.1573	0.026	GDSC
17:40489449-40495449	17:40489449-40495449	CCT007093	-0.1579	0.0237	GDSC
17:40489449-40495449	17:40489449-40495449	BMS-536924	-0.1599	0.0214	GDSC
17:40489449-40495449	17:40489449-40495449	GSK1904529A	-0.1649	0.0183	GDSC
17:40489449-40495449	17:40489449-40495449	Linifanib	-0.1702	0.0128	GDSC
17:40489449-40495449	17:40489449-40495449	SGC0946	-0.1899	0.0052	GDSC
17:40489449-40495449	17:40489449-40495449	JNK Inhibitor VIII	-0.1965	0.0065	GDSC
17:40489449-40495449	17:40489449-40495449	Bicalutamide	-0.2022	0.0027	GDSC

17:40489449-40495449	17:40489449-40495449	BMS-708163	-0.2071	0.0017	GDSC
17:40489449-40495449	17:40489449-40495449	CP724714	-0.2377	0.0002	GDSC
17:40489449-40495449	17:40489449-40495449	Gefitinib	-0.2585	0.0002	GDSC
17:40489449-40495449	17:40489449-40495449	Cetuximab	-0.3145	6.3214e-7	GDSC
17:40489449-40495449	17:40489449-40495449	Afatinib	-0.3253	8.7157e-8	GDSC
17:40489449-40495449	17:40489449-40495449	Afatinib	-0.3476	6.0176e-8	GDSC
