# Supplementary material for: Prognostic Ability of Enhancer RNAs in Metastasis of Non-Small Cell Lung Cancer
Source: Molecules. 2022 Jun 26;27(13):4108. doi: 10.3390/molecules27134108 (PMC9268450; doi:10.3390/molecules27134108)
Supplement: Supplementary file 1 [file molecules-27-04108-s001.zip › Table S2.pdf]

Table S2. The functions of RPPA and their regulatory relationship with PDEE.

PDEE	RPPA	cor	Function
ANXA8L1	PAI1	0.368	promotes glycolysis, thereby promoting the migration and chemotaxis of cancer cells
ANXA8L1	EGFR	0.258	plays an important role in the regulation the proliferation, differentiation, survival, motility, and of the tumor cells
CASTOR2	TFRC	0.318	promotes the proliferation and me-tastasis of cancer cells by upregulating the expression of AXIN2
CYP4B1	NAPSINA	0.365	reported as the specific clinical diagnosis indexes and prog-nostic markers for LUAD
GTF2H2C	TTF1	0.412	reported as the specific clinical diagnosis indexes and prog-nostic markers for LUAD
PSMF1	TFRC	0.251	promotes the proliferation and me-tastasis of cancer cells by upregulating the expression of AXIN2
TNS4	CD49B	0.332	shown as the cell-surface markers for the enrichment of a subpopulation of leiomyoma cells that possess stem/progenitor cell prop-erties
