# Supplementary figures and images for: Prognostic Ability of Enhancer RNAs in Metastasis of Non-Small Cell Lung Cancer
Source: Molecules. 2022 Jun 26;27(13):4108. doi: 10.3390/molecules27134108 (PMC9268450; doi:10.3390/molecules27134108)

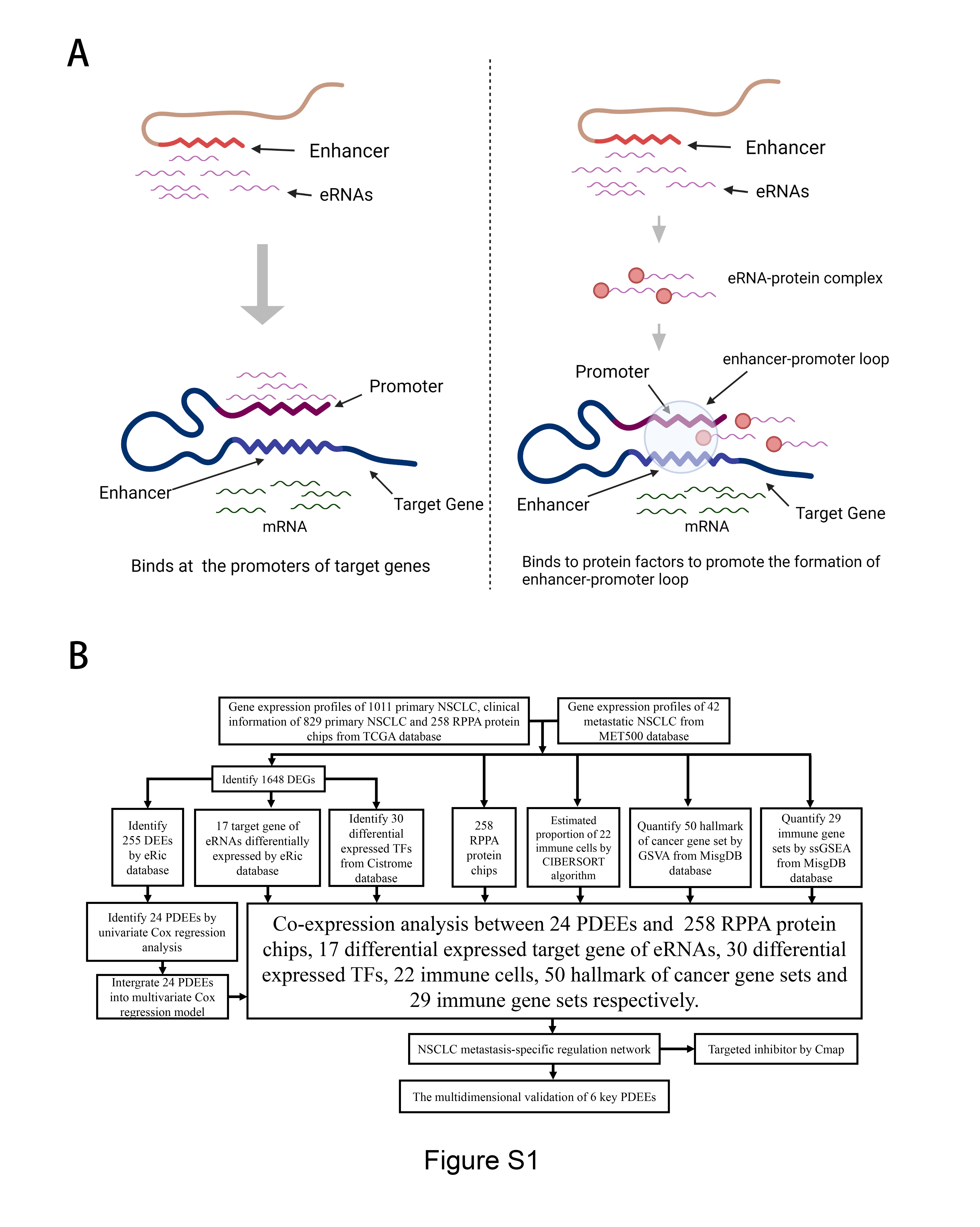

Supplement: Supplementary file 1 [file molecules-27-04108-s001.zip › Figure S1.tif]

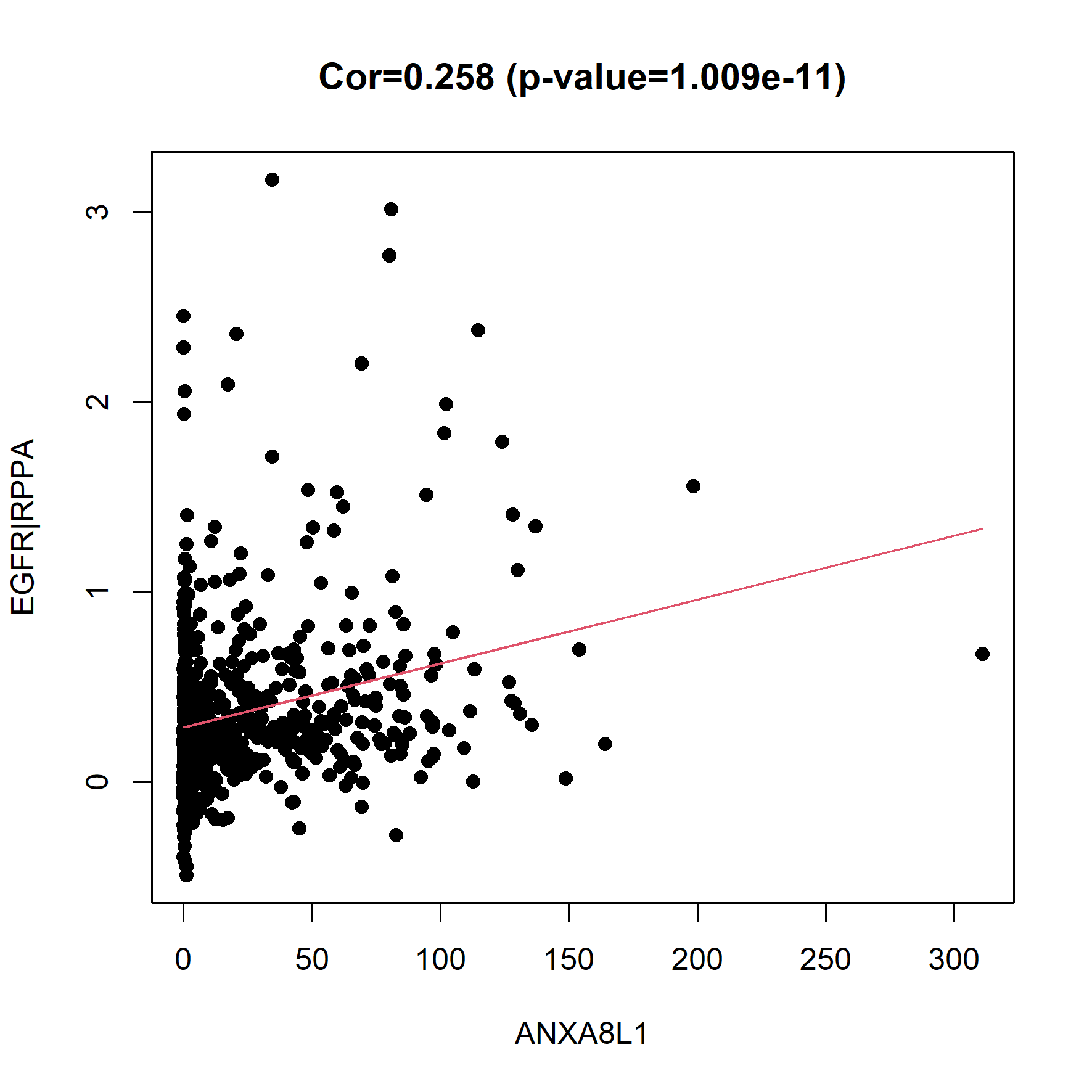

Supplement: Supplementary file 1 [file molecules-27-04108-s001.zip › Figure S20.tif]

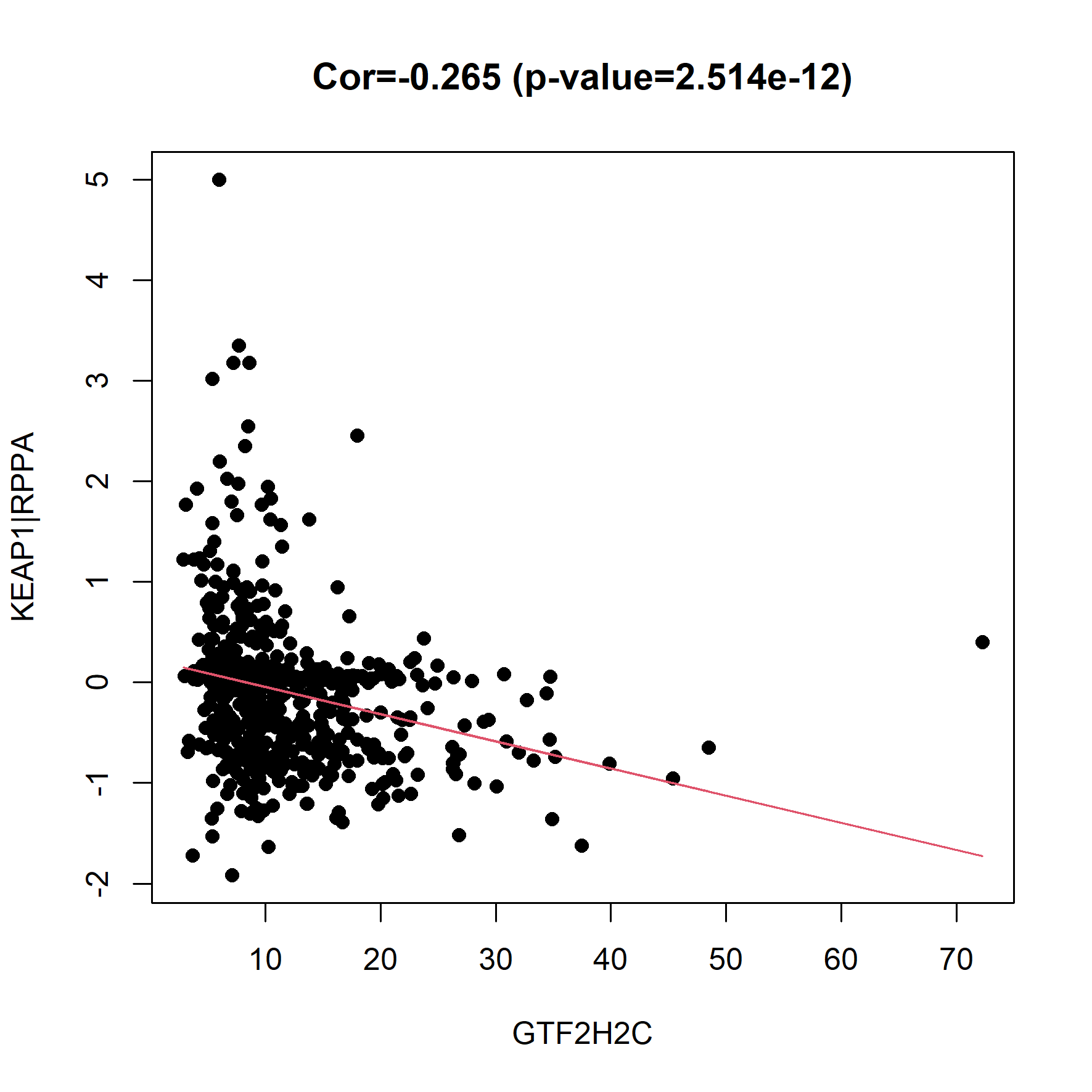

Supplement: Supplementary file 1 [file molecules-27-04108-s001.zip › Figure S21.tif]

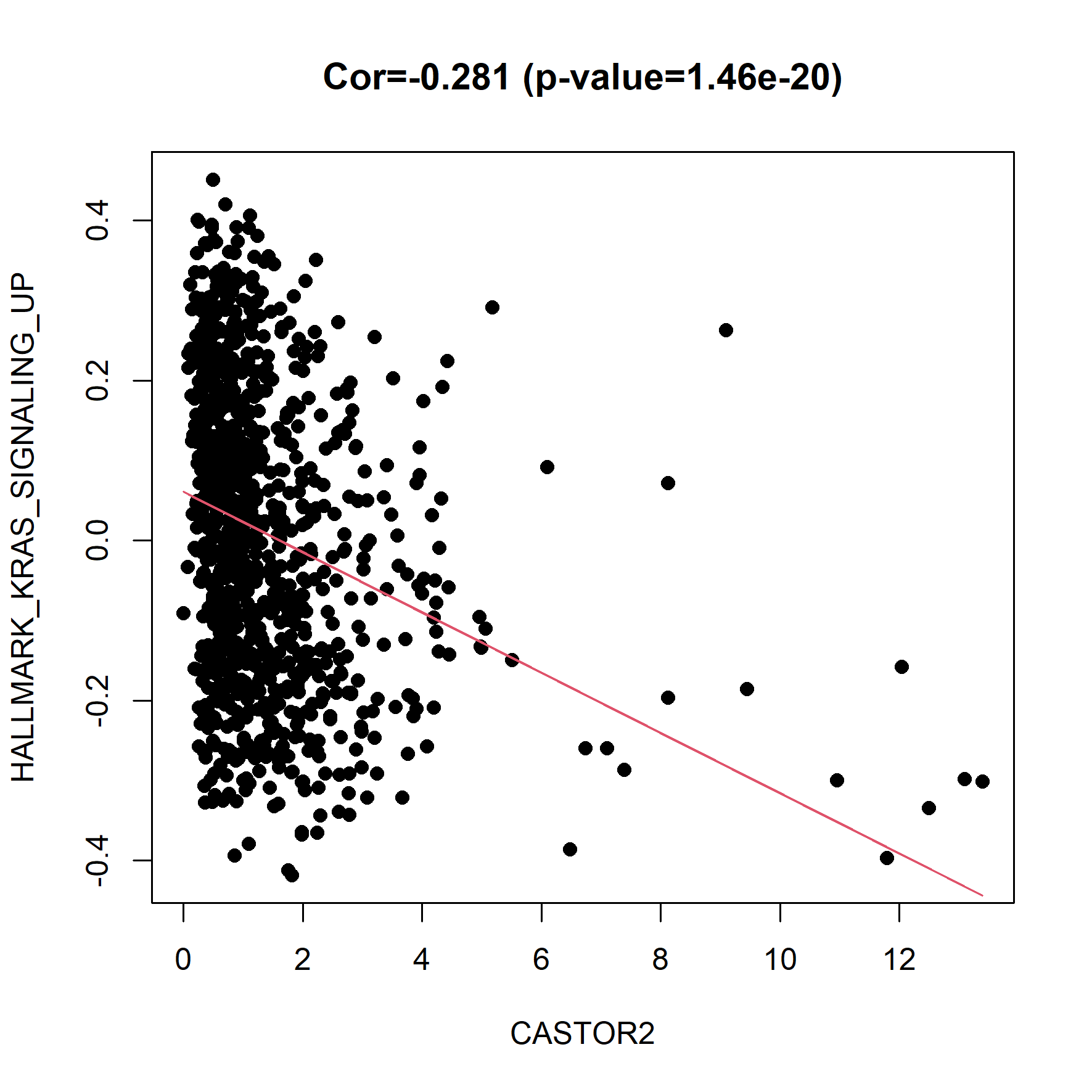

Supplement: Supplementary file 1 [file molecules-27-04108-s001.zip › Figure S22.tif]

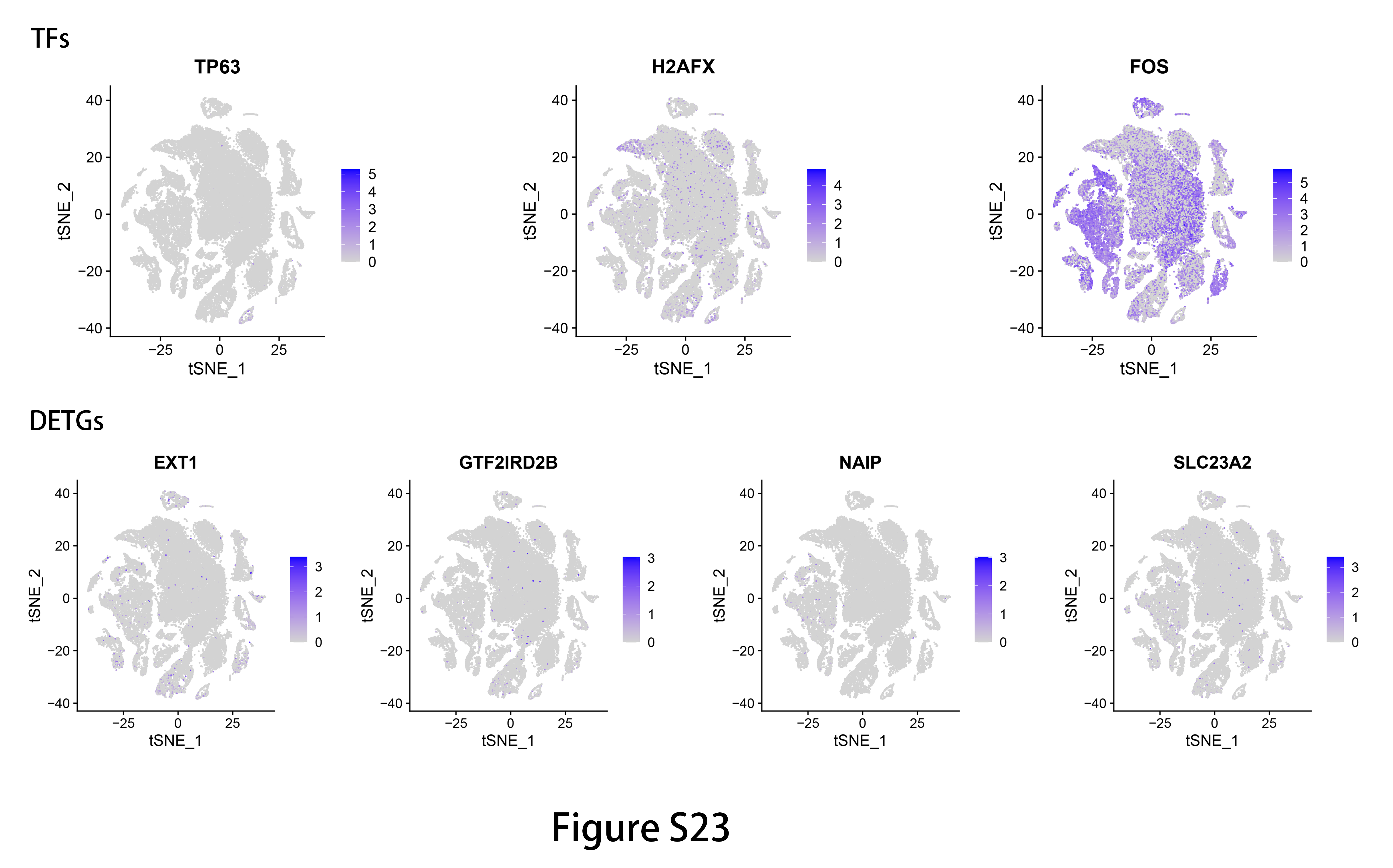

Supplement: Supplementary file 1 [file molecules-27-04108-s001.zip › Figure S23.tif]
